# Supplementary material for: Cell type-specific associations with Alzheimer’s Disease conserved across racial and ethnic groups
Source: bioRxiv. 2025 Apr 15:2025.04.14.648597. Preprint. [Version 1] doi: 10.1101/2025.04.14.648597 (PMC12204241; doi:10.1101/2025.04.14.648597)

bioRxiv preprint doi: https://doi.org/10.1101/2025.04.14.648597; this version posted April 15, 2025. The copyright holder for this preprint (which was not certified by peer review) is the author/funder, who has granted bioRxiv a license to display the preprint in perpetuity. It is made available under aCC-BY 4.0 International license.

Supplemental Tables & Figures

Supplemental Table 1

Effect sizes and p-values of ANCOM-BC and quasibinomial models to assess proportion differences associated with clinical and pathological phenotypes. Columns represent individual population groups as well as the meta-analysis across all three groups (see Methods).

Supplemental Figure 1

Distributions of age (top left) across donor groups, nuclei numbers per cell type per region (top right), total gene counts per nucleus per cell type per region (bottom left), and total transcript (Unique Molecular Identifier) counts per nucleus per cell type per region. For age, the boxplot distribution includes all participants whose brains were profiled, but individual points for those older than 90 have been removed from the visualization.

Supplemental Figure 2

Dotplot showing the fraction of nuclei and mean gene expression in each cluster of broad cell class markers as well as key distinguishing genes between subclusters within each broad class.

Supplemental Figure 3

Compilation of Gene Ontology (GO) terms and aggregated categories for genes differentially expressed within each subcluster versus all other subclusters in the same main class (see Methods).

Supplemental Figure 4

Dotplot showing the fraction of nuclei and mean gene expression in each cluster of the top 5 genes (by loading) on each scHPF-derived factor (see Methods).

Supplemental Figure 5

Compilation of Gene Ontology (GO) terms and aggregated categories for genes with highest loadings on each factor (see Methods).

Supplemental Figure 6

ATAC-seq-based occupancy for key cluster-enriched peaks, with clusters defined based on RNA-seq; this is analogous to Main Figure 3A and 3B. For each major cell class, the heatmap represents the z-score of the mean peak counts per million (pseudobulked over all nuclei of each cluster per sample). For each cluster, the top 2 differential peaks (versus all other clusters of that cell class) are included. Numbers on the right of each heatmap indicate the total # of differential sites per cluster.

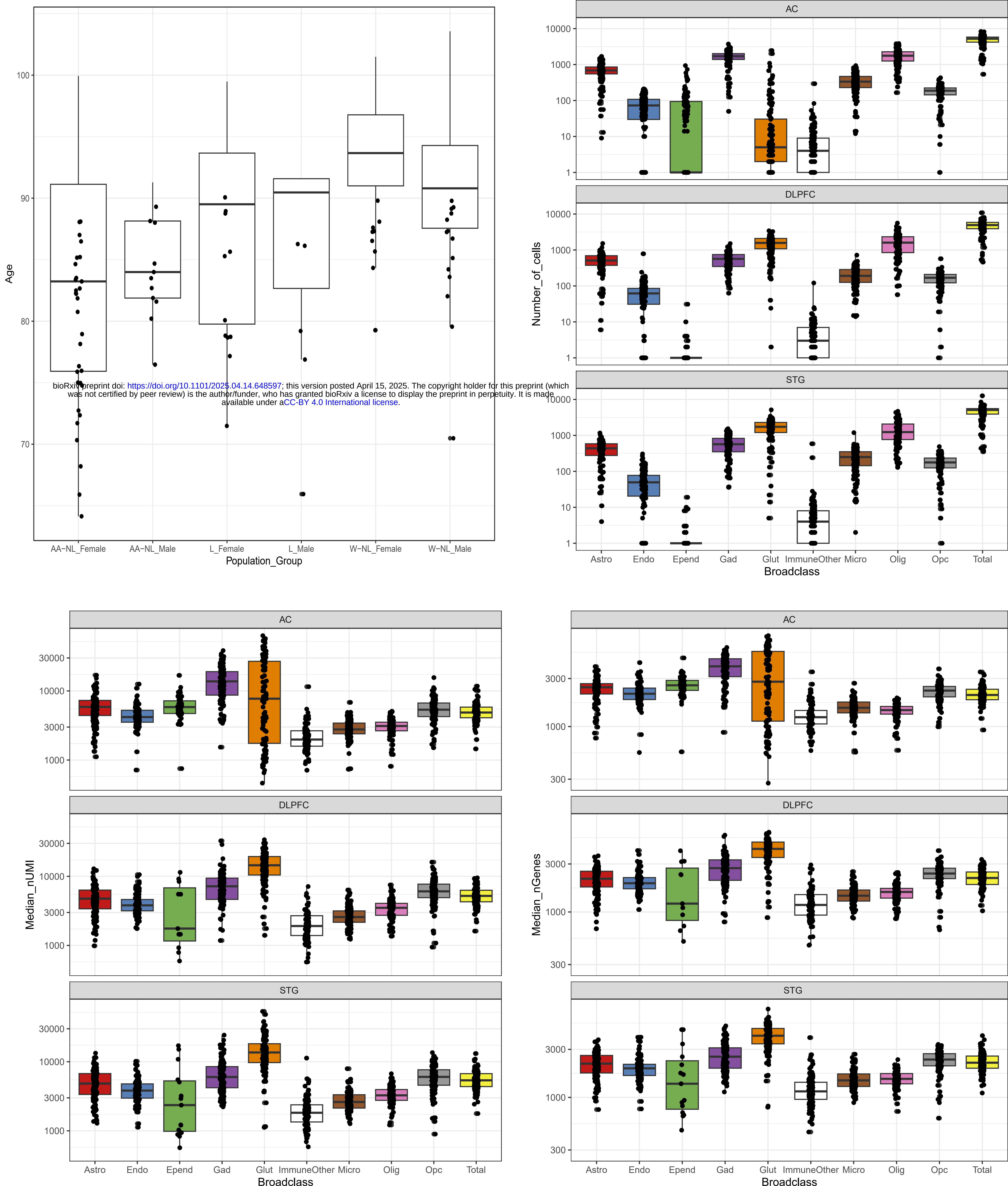

bioRxiv preprint doi: https://doi.org/10.1101/2025.04.14.648597; this version posted April 15, 2025. The copyright holder for this preprint (which was not certified by peer review) is the author/funder, who has granted bioRxiv a license to display the preprint in perpetuity. It is made available under aCC-BY 4.0 International license.

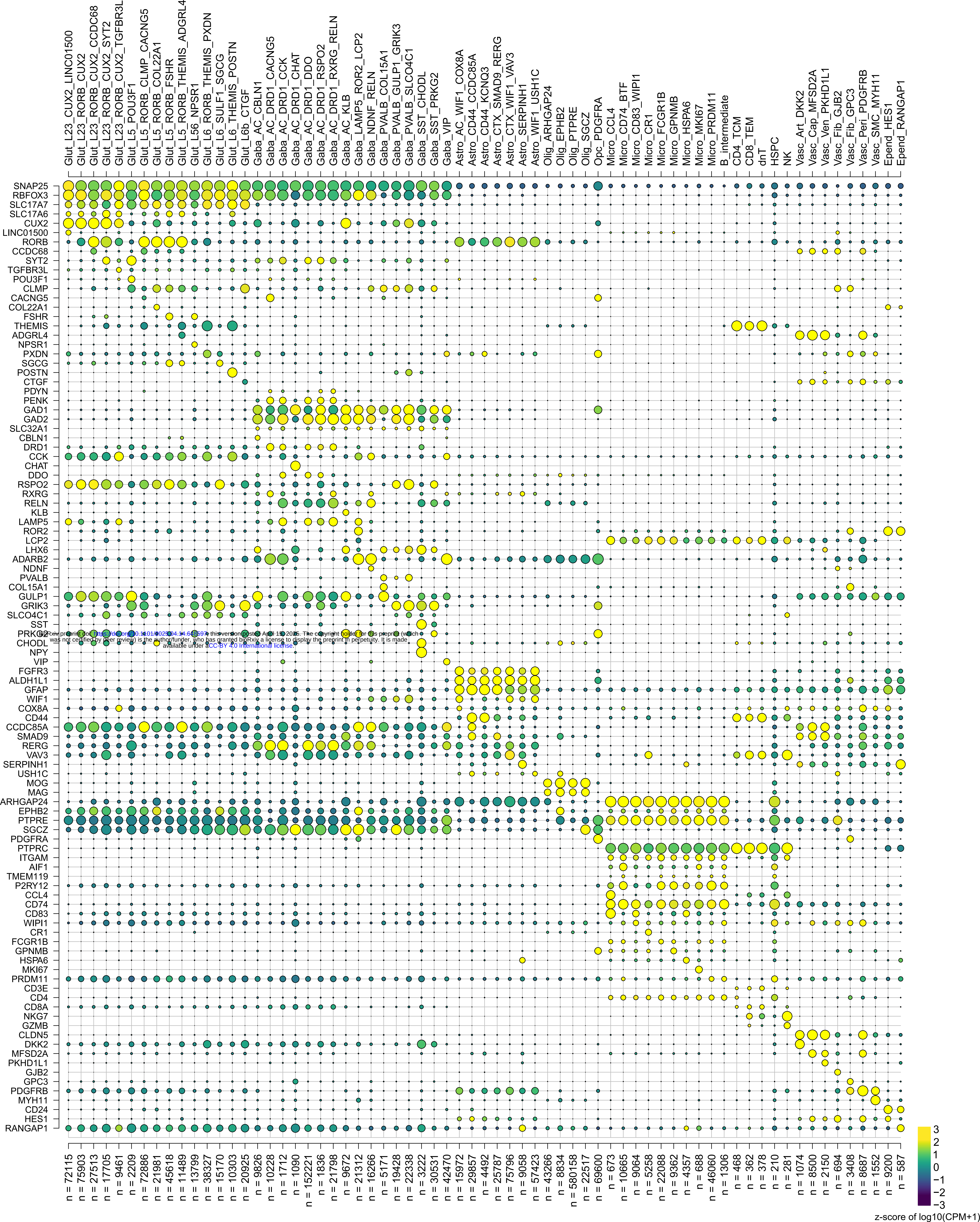

bioRxiv preprint doi: https://doi.org/10.1101/2025.04.14.648597; this version posted April 15, 2025. The copyright holder for this preprint (which was not certified by peer review) is the author/funder, who has granted bioRxiv a license to display the preprint in perpetuity. It is made available under aCC-BY 4.0 International license.

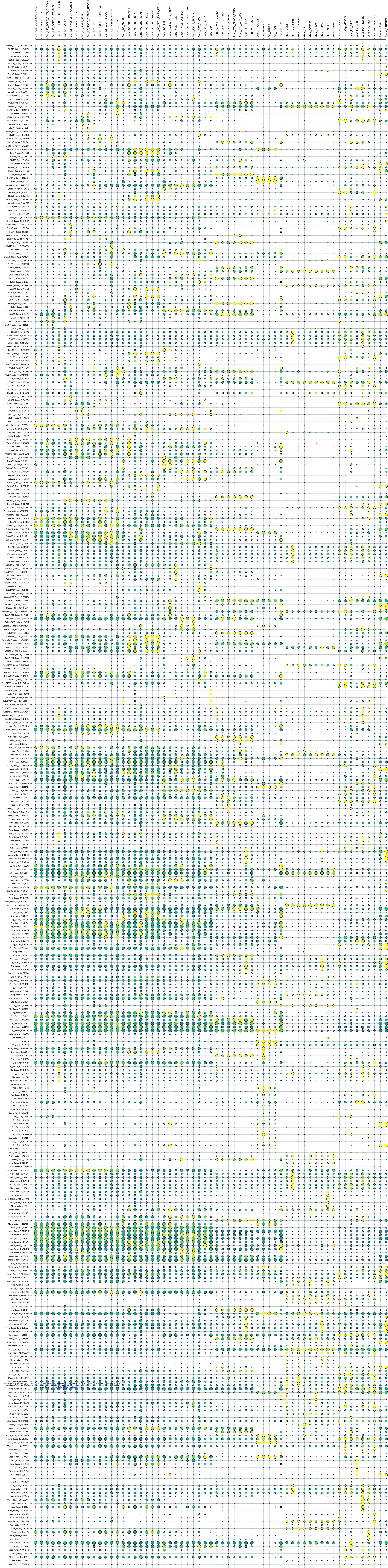

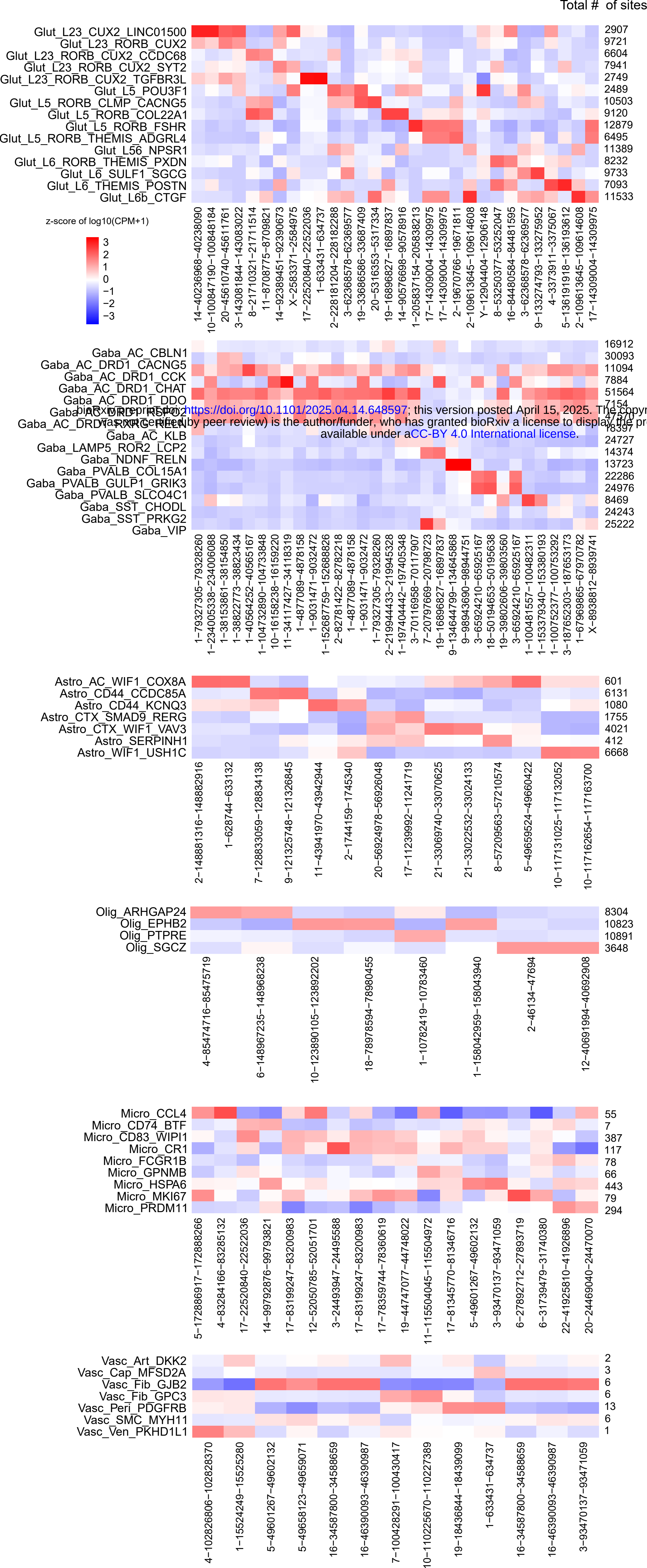

bioRxiv preprint doi: https://doi.org/10.1101/2025.04.14.648597; this version posted April 15, 2025. The copyr[illegible]
was not certified by peer review) is the author/funder, who has granted bioRxiv a license to display the pr[illegible]
available under aCC-BY 4.0 International license.

bioRxiv preprint doi: https://doi.org/10.1101/2025.04.14.648597; this version posted April 15, 2025. The copyright holder for this preprint (which was not certified by peer review) is the author/funder, who has granted bioRxiv a license to display the preprint in perpetuity. It is made available under aCC-BY 4.0 International license.

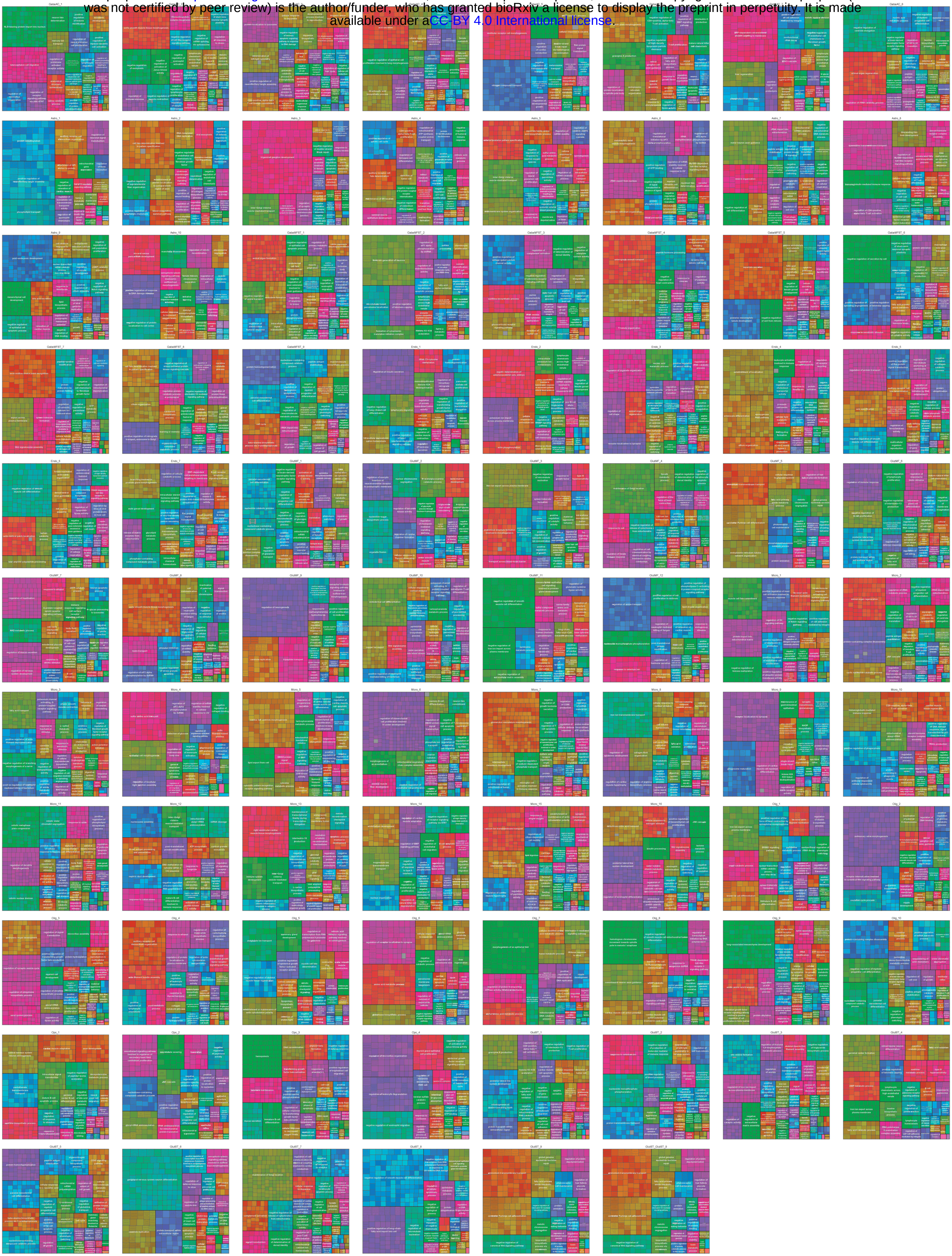

bioRxiv preprint doi: https://doi.org/10.1101/2025.04.14.648597; this version posted April 15, 2025. The copyright holder for this preprint (which was not certified by peer review) is the author/funder, who has granted bioRxiv a license to display the preprint in perpetuity. It is made available under aCC-BY 4.0 International license.

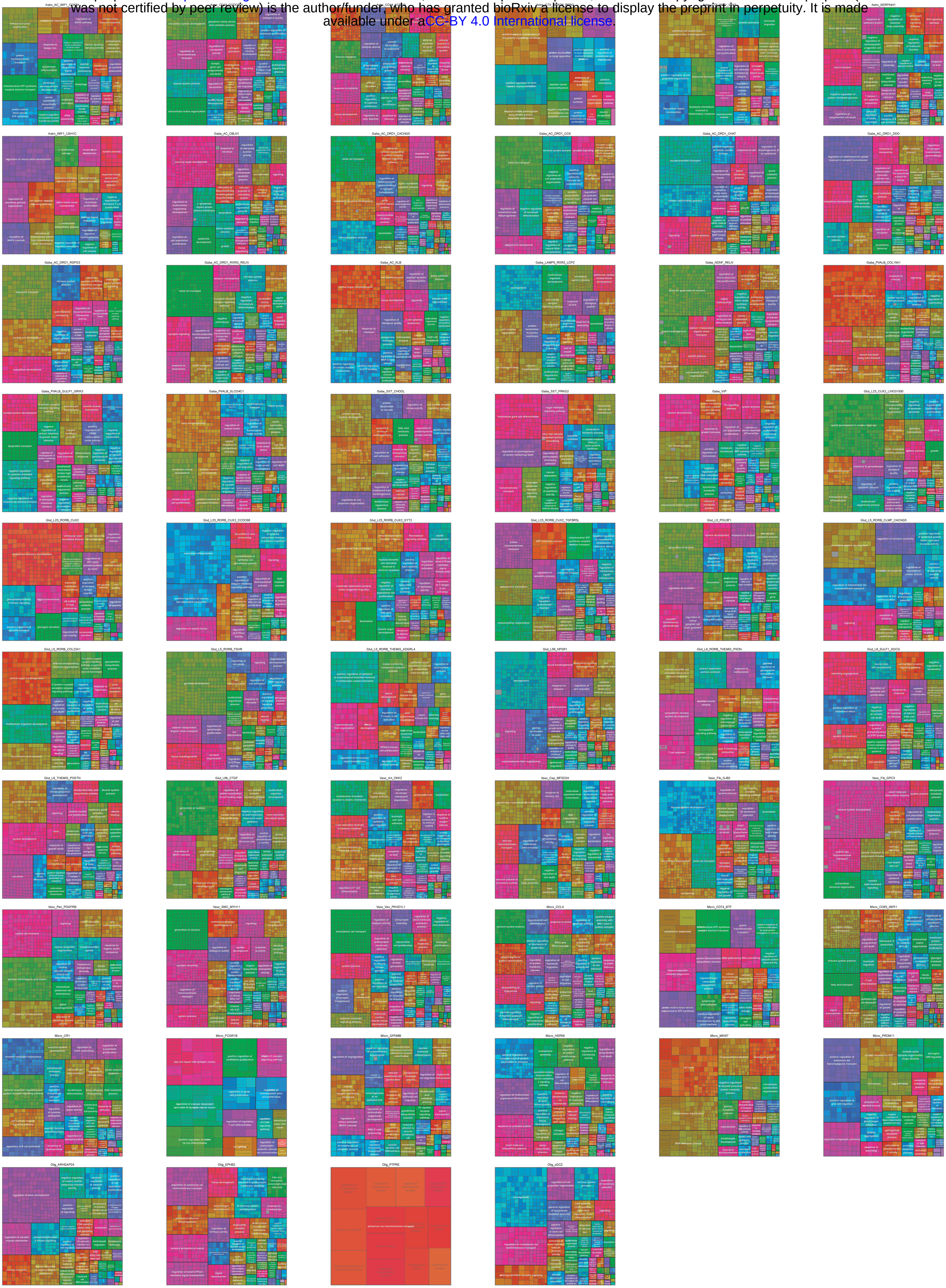

Supplement: 2 [file NIHPP2025.04.14.648597v1-supplement-2.pdf]
